# Optimization and single-laboratory validation of a method for the determination of flavonolignans in milk thistle seeds by high-performance liquid chromatography with ultraviolet detection

**DOI:** 10.1007/s00216-015-8925-6

**Published:** 2015-07-31

**Authors:** Elizabeth Mudge, Lori Paley, Andreas Schieber, Paula N. Brown

**Affiliations:** Natural Health & Food Products Research Group, British Columbia Institute of Technology, 3700 Willingdon Avenue, Burnaby, BC V5G 3H2 Canada; 4-10 Agriculture/Forestry Centre, Department of Agricultural, Food and Nutritional Science, University of Alberta, Edmonton, AB T6G 2P5 Canada; Institute of Nutritional and Food Sciences, Chair of Food Technology and Food Biotechnology, University of Bonn, Roemerstrasse 164, D-53117 Bonn, Germany

**Keywords:** *Silybum marianum* (L.) Gaertn, Flavonolignans, Silymarins, Dietary supplements, High-performance liquid chromatography, Validation

## Abstract

Seeds of milk thistle, *Silybum marianum* (L.) Gaertn., are used for treatment and prevention of liver disorders and were identified as a high priority ingredient requiring a validated analytical method. An AOAC International expert panel reviewed existing methods and made recommendations concerning method optimization prior to validation. A series of extraction and separation studies were undertaken on the selected method for determining flavonolignans from milk thistle seeds and finished products to address the review panel recommendations. Once optimized, a single-laboratory validation study was conducted. The method was assessed for repeatability, accuracy, selectivity, LOD, LOQ, analyte stability, and linearity. Flavonolignan content ranged from 1.40 to 52.86 % in raw materials and dry finished products and ranged from 36.16 to 1570.7 μg/mL in liquid tinctures. Repeatability for the individual flavonolignans in raw materials and finished products ranged from 1.03 to 9.88 % RSD_r_, with HorRat values between 0.21 and 1.55. Calibration curves for all flavonolignan concentrations had correlation coefficients of >99.8 %. The LODs for the flavonolignans ranged from 0.20 to 0.48 μg/mL at 288 nm. Based on the results of this single-laboratory validation, this method is suitable for the quantitation of the six major flavonolignans in milk thistle raw materials and finished products, as well as multicomponent products containing dandelion, schizandra berry, and artichoke extracts. It is recommended that this method be adopted as First Action Official Method status by AOAC International.

## Introduction

**Fig. 1:**
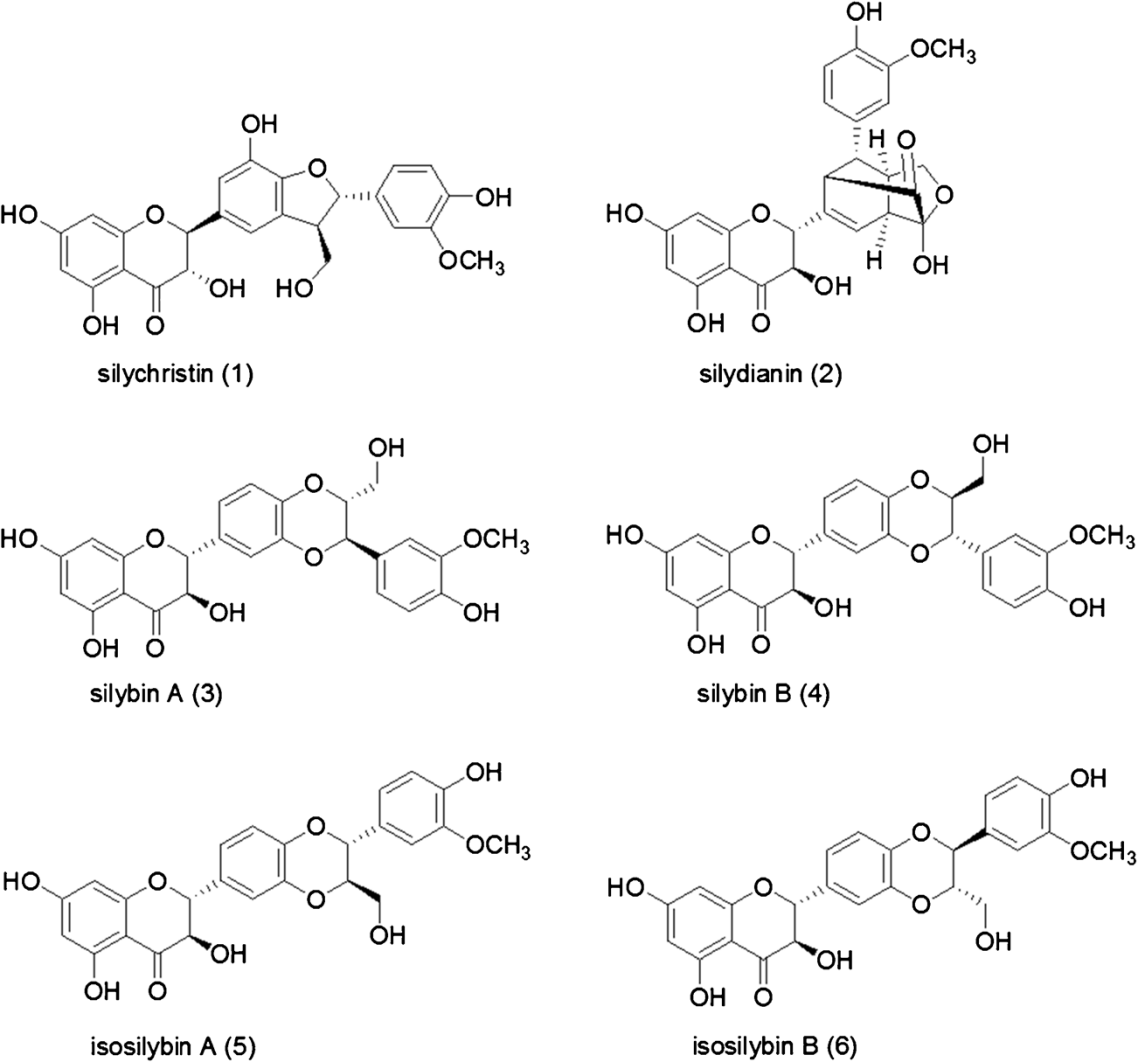
Structure of silychristin (*1*), silydianin (*2*), silybin A (*3*), silybin B (*4*), isosilybin A (*5*), and isosilybin B (*6*)

Herbal dietary supplements, such as milk thistle (*Silybum marianum* (L.) Gaertn.), have considerable variation due to several factors including the quality of starting materials, process conditions, product formulations, and storage. Therefore, stringent analytical methods to evaluate and confirm product quality for both starting materials and finished products are necessary. Milk thistle supplements have consistently been ranked as a top selling product in the past decade and will likely continue as research accumulates in support of its use as a hepatoprotectant [1, 2]. The seeds, which are used in herbal preparations, contain the group of components classified as the silymarins, which have been shown to have hepatoprotective, antioxidant, neuroprotective, antidiabetic, and anti-cancer activities [3–11]. The silymarins are composed of the six major flavonolignans: silychristin, silydianin, silybin A, silybin B, isosilybin A, and isosilybin B shown in Fig. 1.

Milk thistle is available in a variety of formulations including capsules, tablets, tinctures, and dry powders, while other formulations such as phytosomes and liposomes have been developed to improve flavonolignan bioavailability [12, 13]. Products have been enhanced by the addition of several other extracts including schizandra berry, artichoke, and dandelion which may potentially impact flavonolignan quantitation. Traditionally, flavonolignans are determined using HPLC-UV with phosphoric acid in the mobile phase, which excludes the ability to couple the analyses for mass spectrometric detection [14–16]. A growing trend for methods using UPLC and other higher pressure LC systems for separation of flavonolignans has recently occurred, which reduces analysis time and overall solvent usage [17]. As the incorporation of such methods would require many herbal product manufacturers to purchase these new and expensive instruments, they may not be suitable for ubiquitous product quality analysis of milk thistle. Another method that manufacturers use to reduce analysis time is with a non-specific spectrometric method for quantifying total silymarin content, which may lead to erroneous values as many components in the extract may absorb at the specified wavelength.

Clinical evidence of milk thistle efficacy as a hepatoprotectant and in the treatment of liver damage, disease, and cirrhosis has been inconclusive [18–23]. Meta-analyses have pointed out several flaws in study designs including the lack of chemical characterization of products evaluated and the variable dosages administered. To further complicate milk thistle research, clinical trials refer to milk thistle as silibinin, silymarin, or milk thistle, which are all separate entities and are not clearly defined between publications [24]. Inclusion of chemical characterization of products used in milk thistle research is essential in order to evaluate its effectiveness and may lead to more conclusive findings. Therefore, it is essential to develop fast, reliable, precise, accurate, and validated methods for individual flavonolignans in milk thistle raw materials and products.

Milk thistle has been classified as a high priority dietary supplement requiring a validated method for quantifying flavonolignan content. In 2009, an AOAC Expert Review Panel (ERP) convened to evaluate methods submitted for milk thistle quantitation and determined the INA method 115.00 Silymarins in milk thistle by HPLC was suitable; provided several modifications are evaluated prior to validation [15, 25]. The ERP recommended several modifications to the original method. This method involved defatting of the milk thistle seeds for several hours using a Soxhlet extractor, followed by an overnight methanol Soxhlet extraction of the flavonolignans [15]. One recommendation of the ERP was to evaluate the necessity of the defatting step and also to adapt the method for multiple matrices. Other recommendations included evaluating mobile phases compatible with both ultraviolet and mass spectrometric detectors and improving the chromatographic separation [25].

The objectives of this work were to address the modifications suggested by the ERP on milk thistle method optimization by using statistically guided optimization procedures. Several extraction parameters were evaluated including pretreatment volume, contact time, and extraction method. The optimized method for flavonolignans in milk thistle was subjected to a single-laboratory validation according to AOAC International guidelines [26]. The method quantifies the six major flavonolignans in several matrixes including raw milk thistle seeds, bulk extract, single and multi-component capsules, tablets, and tinctures.

## Experimental

### Reagents and reference materials

HPLC grade methanol was purchased from VWR International (Mississauga, ON, Canada). HPLC grade hexane and ACS grade formic acid and sulfuric acid were purchased from Fisher Scientific (Ottawa, ON, Canada). Water was deionized using a Barnstead water purification system from Fischer Scientific and further filtered through 0.22 μm nylon filters. The high-purity certified reference materials silybin A, silybin B, silychristin, silydianin, isosilybin A, and isosilybin B were purchased from Cerilliant Corporation (Round Rock, TX). A silybin A/B combination standard was provided from Chromadex (Irvine, CA). All standards were stored in a desiccator at −20 °C for long-term storage.

### Purity assessment of reference materials

To confirm purity, quantitative NMR (qNMR) was performed on the individual flavonolignan reference standards using a Varian Mercury VX spectrometer operating at 400.13 MHz for ^1^H. Samples were dissolved in deuterated DMSO and analyzed by standard proton NMR spectrometry for identification and qNMR for quantitative analysis according to Pauli et al. [27]. The silybin A/B mixture standard was assessed for purity by calibrating the concentrations using the individual flavonolignan standards.

### Test materials

One source of milk thistle seeds was used in the optimization studies. This source was cultivated in 2009 at Midmore Organic Farm (Morinville, AB, Canada) and deposited in the University of Alberta Vascular Plant Herbarium, accession number ALTA 126811, under the supervision of botanist Dorothy Fabijan. Eleven samples were used in the single-laboratory validation. Two sources of *S. marianum* seeds were obtained from commercial suppliers. The first being the one used in the optimization study and the second was purchased from Horizon Herbs (Williams, OR). A milk thistle powdered extract was provided by Euromed (Monza, Italy). Several commercial products were purchased from local health stores. These include single ingredient milk thistle products and multi-component products with extracts such as schizandra berry, dandelion, and artichoke. The test samples are described in Table 1.

**Table 1:** Composition of each test sample subjected to the single-laboratory validation including the dilution required for the samples to be within the calibration range of the method

Sample code	Sample type	Constituents	Dilution factor
MT-RM001	Raw material	Milk thistle seeds	n/d
MT-RM002	Raw material	Milk thistle seeds	n/d
MT-BE001	Bulk extract	Milk thistle extract	1:5
MT-CP001	Hardshell capsule	Milk thistle extract	1:10
MT-CP002	Hardshell capsule	Milk thistle, schizandra berry	1:10
MT-CP003	Hardshell capsule	Milk thistle, dandelion, artichoke extract	1:10
MT-TB001	Tablet	Milk thistle extract, milk thistle seed	n/d
MT-TB002	Tablet	Milk thistle extract	1:10
MT-TN001	Tincture	Milk thistle extract	1:5
MT-TN002	Tincture	Milk thistle, dandelion extract	1:2
MT-TN003	Tincture	Milk thistle extract	1:5

*n/d* no dilution

### HPLC analysis

An Agilent 1290 HPLC system equipped with an autosampler, binary pump, and diode array detector (Agilent Technologies, Mississauga, ON, Canada) was used. The separation was achieved on a Kinetex® XB-C18 2.6 μm, 3.0 × 100 mm column (Phenomenex, Torrance, CA). The mobile phase was composed of (a) 0.1 % formic acid in water and (b) 0.1 % formic acid in 80 % aqueous methanol. The flow rate for the separation was 0.4 mL/min (0 to 36 min, 45.1 to 46 min) and 0.45 mL/min during the re-equilibration (36.1 to 45 min). The gradient elution was as follows: 0–1 min: 15 %B; 1–2 min: 15–43 %B; 2–10 min: 43–45 %B; 10–25 min: 45–55 %B; 25–27 min: 55–60 %B; 27–35 min: 60–100 %B; 35–36 min: 60–100 %B; 36–36.1 min: 100–15 %B; 36.1–45 min: 15 %B. The column temperature was 25 °C and injection volume was 2 μL. UV spectra were collected from 200 to 400 nm with 288 nm used for detecting the flavonolignans. Data was processed using OpenLab software (Agilent Technologies).

### Optimization—pretreatment

#### Hexane defatting

Five 10 g replicates of ground milk thistle seed were weighed into cellulose extraction thimbles and extracted with 100 mL of hexane using a Soxhlet apparatus for 6 h. Thimbles were dried at 40 °C to remove residual solvent.

#### Sulfuric acid pretreatment

Five 4 g replicates of ground milk thistle seed were weighed into 50 mL polypropylene tubes and treated with 40 mL of 1.5 % *v*/*v* sulfuric acid at 50 °C in a water bath shaker at 60 rpm for 24 h. After cooling to room temperature, the samples were centrifuged at 5000 rpm for 5 min and the supernatant was discarded. The solids were recovered and allowed to dry at room temperature for 24 h.

Five 300 mg replicates of control (no pretreatment), hexane-treated, and sulfuric acid-treated material were extracted with 40 mL methanol for 30 min using a sonicating bath at 45 °C. After cooling to room temperature, the samples were centrifuged at 5000 rpm for 5 min. The methanol was transferred to a 50-mL volumetric flask and brought up to volume with methanol. An aliquot was placed into an HPLC vial and analyzed for flavonolignan content by HPLC-UV.

#### Pretreatment contact time

Triplicate 200 mg samples of ground milk thistle seeds were extracted with 10 mL of 1.5 % H_2_SO_4_ in a 50 °C water bath shaking at 60 rpm for 0.5, 1, 2, 4, 6, 18, and 24 h. The samples were cooled and centrifuged at 5000 rpm for 5 min and the supernatant discarded. Samples were extracted with 25 mL of methanol and sonicated at 45 °C for 30 min. They were cooled to room temperature and centrifuged at 5000 rpm for 5 min, and a 1-mL aliquot was analyzed for flavonolignan content by HPLC-UV.

#### Pretreatment and rinse volume

In triplicate, 200 mg samples of ground milk thistle seed were pre-treated with either 10 mL 1.5 % H_2_SO_4_ solution or 2 mL 1.5 % *w*/*w* H_2_SO_4_ solution for 30 min. Samples were cooled, centrifuged at 5000 rpm for 5 min, and decanted and rinsed by adding either 10 or 2 mL respectively of deionized water. After vortexing for 30 s, samples were centrifuged at 5000 rpm for 5 min and the rinse water was discarded. Each sample was extracted with methanol as per the sonication procedure above and analyzed for flavonolignan concentration. These experiments were repeated with the rinse step omitted.

### Optimization—extraction

#### Soxhlet procedure

Five 5 g replicates of defatted ground milk thistle seed were extracted with 90 mL of methanol using a Soxhlet extraction apparatus for 8 h. Extracts were cooled and transferred to 100 mL volumetric flasks and diluted to volume with methanol. One milliliter aliquots were analyzed for flavonolignan concentration. This procedure was repeated with five 1 g replicates of ground milk thistle tablets.

#### Sonication procedure

Five 150 mg (±10 %) replicates of defatted ground milk thistle seed were extracted with 25 mL of methanol using a sonicating bath at 45 °C for 30 min. Samples were cooled to room temperature and centrifuged at 5000 rpm for 5 min, and a sample of the solution was analyzed for flavonolignan concentration. This was repeated with five 70 mg (±10 %) replicates of ground milk thistle tablets.

### Method validation—reference solution preparation

Individual 1000 μg/mL stock solutions of each standard were prepared by weighing 10 mg of each standard into separate 10 mL volumetric flasks and diluted with methanol. Isosilybin A and isosilybin B stock solutions were diluted to 500 μg/mL working stock solutions prior to preparation of the calibration standards each day from the original stock solutions.

The solutions for the calibration curves were prepared using serial dilutions of the stock solutions with methanol. The nominal concentrations of each standard in the seven-point calibration curves are summarized in Table 2.

**Table 2:** Nominal concentrations of the individual flavonolignans in each of the calibration solutions

Flavonolignan	Approximate concentration (μg/mL)							Average correlation coefficients (*r* ^2^)
	Lin 1	Lin 2	Lin 3	Lin 4	Lin 5	Lin 6	Lin 7	
Silychristin	150	100	75	50	15	5	1.5	0.9992
Silydianin	150	100	75	50	15	5	1.5	0.9994
Silybin A	150	100	75	50	15	5	1.5	0.9992
Silybin B	150	100	75	50	15	5	1.5	0.9994
Isosilybin A	75	50	35	20	7.5	2	0.75	0.9993
Isosilybin B	75	50	35	20	7.5	2	0.75	0.9991

### Method validation—sample preparation

#### Raw materials

Milk thistle seeds were ground using a water-jacketed hammer mill or grinder to <40 mesh powder. Test samples (200.0 mg, ±5.0 mg) were weighed into a 50-mL conical tube and 2.0 mL of 1.5 % H_2_SO_4_ was added. Samples were vortexed for 30 s and placed in a 50 °C shaking water bath at 60 rpm for 30 min. After the samples were cooled to room temperature, they were centrifuged at 5000 rpm for 5 min. The pretreatment solution was decanted and 2 mL of rinse water was added. Samples were vortexed for 30 s and centrifuged at 5000 rpm for 5 min. The rinse solution was decanted to waste. Twenty-five milliliters of 100 % methanol was added to each sample and vortexed for 30 s. The flavonolignans were extracted for 30 min in a heated sonicating water bath at 45 °C. Samples were cooled to room temperature and centrifuged at 5000 rpm for 5 min. An aliquot of the extract was filtered using a 0.45-μm Teflon filter in an HPLC vial and subjected to HPLC analysis.

#### Powdered extracts, capsules, and tablets

The contents of the 20 capsules were emptied and combined in a conical tube. Weights of the contents and empty shells were obtained, and the average fill weight was recorded. Contents were mixed using a spatula to homogenize the samples. Twenty tablets/caplets were combined, weighed, and ground using a coffee grinder. Test material (100.0 mg, ±5.0 mg) was weighed into a 50-mL conical tube and 25 mL of methanol was added using a volumetric pipet. The samples were vortexed and extracted in a heated sonicating water bath at 45 °C for 30 min. Samples were cooled to room temperature and centrifuged at 5000 rpm for 5 min.

Samples which are outside the calibration range were diluted either 1:5 or 1:10 with methanol prior to filtration. The dilution factors for each sample are summarized in Table 1. All samples were filtered using 0.45 μm Teflon filters into HPLC vials and analyzed by HPLC.

#### Tinctures

Tinctures were mixed thoroughly by inversion and diluted 1:2 or 1:5 with methanol and vortexed for 30 s. The dilution factors for each sample are summarized in Table 1. The diluted samples were filtered using a 0.45-μm Teflon filter into an HPLC vial and analyzed by HPLC. Note: Dilution of at least 1:2 is required as the high water content in tinctures causes solubility issues during filtration.

### Method validation

The above method was validated according to AOAC International guidelines for conducting single-laboratory validation [26].

#### Limits of detection (LOD) and quantitation (LOQ)

Suitable matrix blank was not available; therefore, the use of the International Union for Pure and Applied Chemistry method for determination of detection limits was not possible. The LOD for each analyte was determined using the US Environmental Protection Agency Method Detection Limit (MDL) protocol [28]. The MDL is defined as the minimum concentration of substance that can be measured and reported with 99 % confidence that the analyte concentration is greater than zero. To ensure matrix effects are still present, the tincture MT-TN003 was diluted so that all flavonolignans were at a very low concentration. Seven replicates were injected and the calculation of the MDL was as follows:

$$ \mathrm{M}\mathrm{D}\mathrm{L}=s\times {t}_{\left(0.01,n-1\right)} $$

Where *s* is the sample standard deviation of the replicates and t_(0.01, *n*-1)_ is the *t* statistic with *α* = 0.01 and *n* − 1 degrees of freedom.

A second set of seven replicates were assessed by diluting the tincture to another low concentration. This was performed to ensure variance is consistent at the low concentrations and to confirm that the MDLs are valid.

The LOQ was calculated as 10 times the sample standard deviation of the results for the replicates used to determine the MDL.

#### Precision

Twelve replicates were analyzed for each test sample. Four replicate samples of each material were prepared and analyzed on three separate days. The within-day, between-day, and total standard deviation values were calculated for each of the individual flavonolignans. The HorRat value for each flavonolignan in each material was also calculated to assess the overall precision of the method as described by Horwitz [29].

#### Accuracy

One hundred milligrams of negative control material, composed of 99 % maltodextrin and 1 % magnesium stearate, was spiked with reference standards to contain total flavonolignan contents of 1.5, 5.0, and 11.8 % *w*/*w* and diluted to a total volume of 25 mL with methanol, followed by sonication for 30 min at 45 °C. Samples were prepared in triplicate on three separate days and subjected to HPLC analysis.

#### Stability of standards

Flavonolignan stability was assessed by preparing a stock solution containing 100 ppm of each flavonolignans in methanol and was stored at room temperature for 72 h. Aliquots of the solution were analyzed in triplicate at the time points 0, 8, 24, 48, and 72 h. Peak areas were compared to time zero and deviations less than 5 % were considered acceptable.

### Data analysis

Individual flavonolignans from solid samples were quantified in % (*w*/*w*) and liquid samples were quantified in μg/mL using external calibration. Microsoft Excel was used for calculations and statistical analyses. Optimization data was evaluated with single-factor analysis of variance (ANOVA) to determine whether statistically significant differences exist between data sets. Where appropriate, Tukey’s Honestly Significant Difference (HSD) post hoc test was used to establish the significance of results. HorRat values were also calculated using Microsoft excel. The calculations used to determine the Horwitz ratio (HorRat), a normalized performance parameter used to evaluate overall method precision, are provided below:

$$ {\mathrm{RSD}}_{\mathrm{r}}\left(\mathrm{found},\%\right):{\mathrm{RSD}}_{\mathrm{r}}=\frac{\mathrm{SD}\left(\mathrm{R}\right)}{\mathrm{mean}}\times 100 $$

Where SD(r) is the population SD (σ/*n*, where *σ* is the sum of squares and *n* is the number of replicates).

$$ {\mathrm{PRSD}}_{\mathrm{r}}\left({\mathrm{RSD}}_{\mathrm{r}}\mathrm{calculated},\%\right):{\mathrm{PRSD}}_{\mathrm{r}}=2{\mathrm{C}}^{\hbox{-} 0.15} $$

Where C is the concentration of the analyte expressed as a mass fraction.

$$ \mathrm{HorRat}\kern0.5em \mathrm{value}:\mathrm{HorRat}\frac{{\mathrm{RSD}}_{\mathrm{r}}}{{\mathrm{PRSD}}_{\mathrm{r}}} $$

## Results and discussion

### Chromatographic optimization

**Fig. 2:**
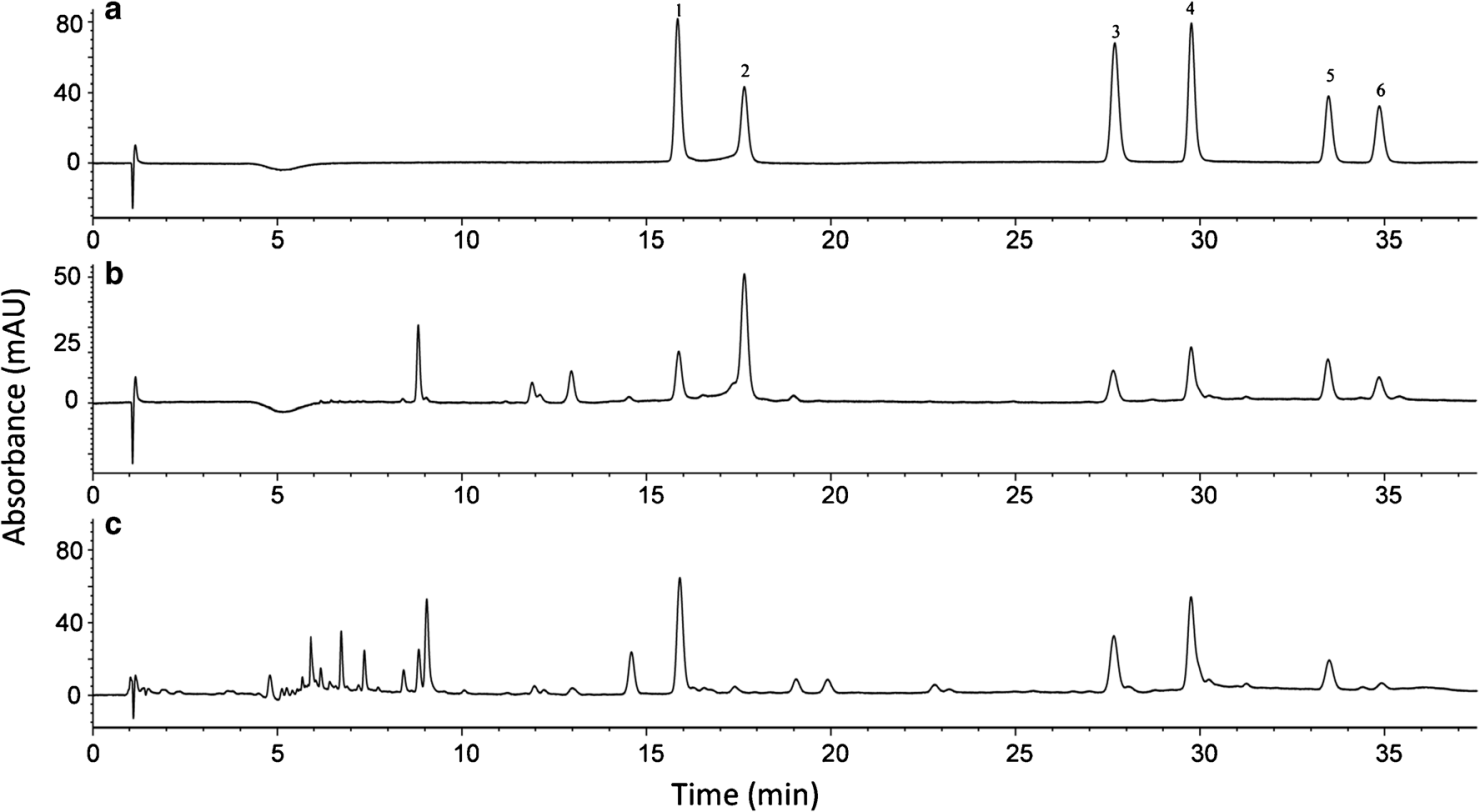
HPLC separation and identification of flavonolignans in mixed flavonolignan reference standard (**a**), milk thistle seeds (**b**), and milk thistle tincture (**c**), all in methanol at 288 nm

New technologies in column manufacture using core-shell packing allows for the development of faster and/or higher resolution separations without requiring new, higher pressure LC systems. Optimization of the HPLC separation of silymarins was evaluated using several columns including Kinetex® 2.6 μm, 3.0 × 100 mm C8, C18, and XB-C18 core shell columns, where the XB-C18 column was chosen for further optimization. Using a mixture of the six flavonolignan standards, the separation was achieved in less than 25 min (results not shown). When used to analyze the commercial products and milk thistle seeds, it was evident that several minor components were co-eluting with the silychristin, silydianin, and silybin B peaks. The separation was increased to 46 min using the milk thistle tincture to optimize the resolution. The chromatograms of the standard mixture, the milk thistle seeds, and the tincture are shown in Fig. 2. Due to the complexity of natural products, including milk thistle extracts, baseline resolution is rarely possible for all major analytes. As previously observed, there are several minor isomers that co-elute with silybin B with the same molecular weight and are not resolved in traditional HPLC methods [30]. Therefore, this method improves the separation of these minor components from the flavonolignans of interest. It was observed that with MS compatible solvents, silydianin has some peak fronting, which was improved using the shell core XB-C18 column compared to other columns [30].

The column used in this separation provided several benefits including a reduction in solvent consumption, increased resolution of the flavonolignans, and a shorter run time compared to the original INA method [15]. The low back pressure from this column permits this method to be used by quality control labs equipped with traditional HPLC systems. This provides significant cost savings in the separation alone when this method is incorporated into laboratories.

### Optimization studies

#### Pretreatment

Milk thistle seeds can contain as much as 26–31 % fat, which interferes with flavonolignan extraction [15, 31, 32]. The traditional Soxhlet method involves long extraction times, high temperatures, large sample sizes, and high solvent consumption per sample, which are not suitable for fast routine methods. Pretreatment of milk thistle seeds with 1.5 % H_2_SO_4_ is a potential alternative to replace defatting and has been shown to improve yields in bulk extractions [33]. The dilute acid was thought to break the seed coat cells, allowing for efficient extraction of the phytochemicals, while not interfering with the chemical composition of the flavonolignans [33]. In this work, optimization procedures for quick, routine analysis were guided using statistical analysis based on the total silymarin levels.

**Fig. 3:**
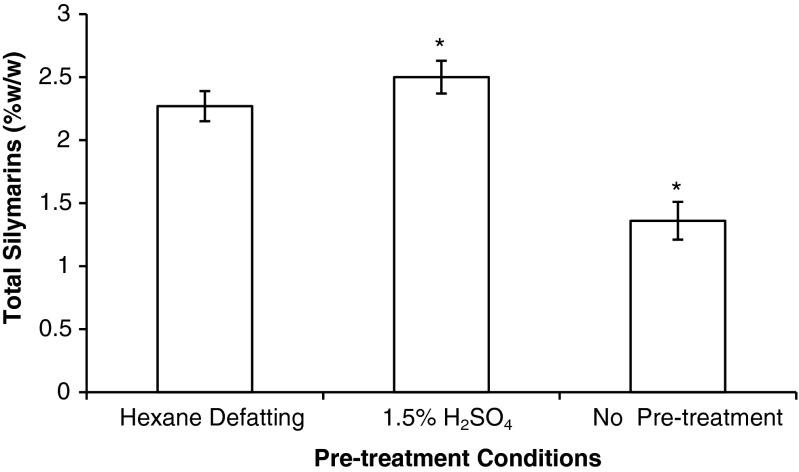
Comparison of silymarin yield in milk thistle seeds after three different pre-treatment methods. *Asterisk* signifies significance (*p* < 0.05) from the hexane defatting pretreatment condition

The pretreatment studies evaluated the silymarin yields after hexane defatting, treatment with 1.5 % H_2_SO_4_, and an untreated control. These results are summarized in Fig. 3, confirming that a pretreatment step is necessary prior to the silymarin extraction. There are significant differences between the three pretreatment groups (F_(2,12)_ = 103, *p* < 0.001). A Tukey’s HSD post hoc test demonstrated that the hexane pretreatment is significantly different from the control (non-pretreated) and acid pretreated samples with a q_obs_ (15.2) and (−3.98) > q_crit_ (3.15). As the acid pretreatment uses more environmentally friendly reagents, less glassware, lab space, and equipment, it was further optimized. Several pre-treatment solutions were analyzed for by HPLC, confirming that the flavonolignans were not extracted during pre-treatment.

In previous work, the exposure time was 24 h for pretreatment [33]. This long pretreatment is not suitable for fast sample preparation, so the exposure time varied from 0.5 to 24 h. As summarized in Table 3, there are significant differences between the 7 pretreatment times (F_(6,14)_ = 24.6, *p* < 0.001). Treatment times (0.5, 1, 2, and 4 h) were found to be significantly higher than 24 h, given that q_obs_ > q_crit_ for all. The flavonolignan levels decreased over time when exposed to high temperature solvents; therefore, in order to reduce the exposure of the samples to heat, the 0.5-h pre-treatment time was selected. No significant differences were observed for pretreatment or rinse volumes (F_(3,8)_ = 1.02, *p* = 0.434). Therefore, 2 mL was chosen to reduce solvent use.

**Table 3:** Comparison of total silymarin content in milk thistle seeds after pretreatment exposure times from 0.5 to 24 h with 1.5 % H_2_SO_4_

Pretreatment time (h)	Average (%*w*/*w*)	Standard deviation (%*w*/*w*)	ANOVA		HSD post hoc test
			Variance	*P* value	
0.5	2.15	0.06	0.004	<0.001	11.9^a^
1	2.13	0.09	0.008		11.2^a^
2	2.05	0.07	0.005		9.01^a^
4	2.02	0.05	0.002		7.92^a^
6	1.84	0.05	0.003		2.59
18	1.78	0.02	0.0003		0.90
24	1.75	0.03	0.0008		–

^a^Significance at *α* = 0.05 compared with 24 h pretreatment time

#### Extraction

**Fig. 4:**
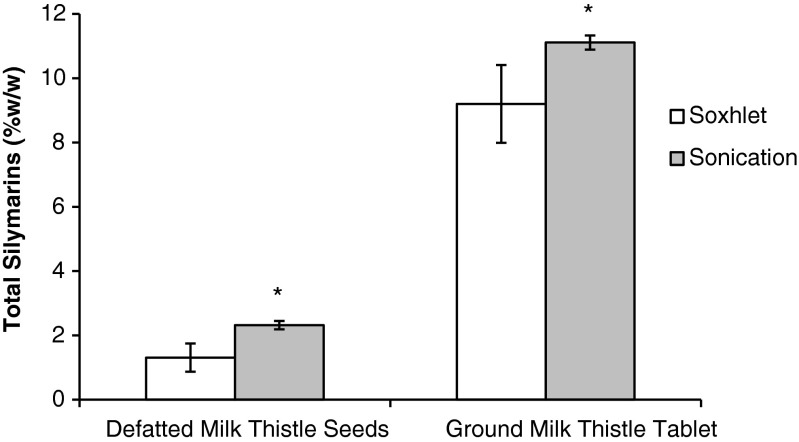
Comparison of silymarin content (%*w*/*w*) in defatted milk thistle seeds and in ground milk thistle tablets using two extraction techniques: Soxhlet and sonication at 45 °C. *Asterisk* signifies significance (*p* < 0.05) from the Soxhlet extraction technique

It is necessary to evaluate if sonication used in the pretreatment optimization is a comparable alternative to the industry standard Soxhlet extraction. The two extraction methods were compared using defatted milk thistle seed and a milk thistle tablet. The silymarin content in the seeds and tablet were significantly higher using sonication compared with Soxhlet (F_(1,8)_ = 24.6, *p* <0.01; F_(1,8)_ = 12.0, *p* <0.01), as shown in Fig. 4. Both materials also resulted in lower variance using sonication. Therefore, sonication is a suitable alternative extraction method of the flavonolignans compared to Soxhlet extraction. When evaluating the individual flavonolignan contents, silydianin levels were lower with Soxhlet extraction. Silydianin levels decrease significantly when stored as tinctures at room temperature, indicating that it is possibly the least stable of the six major flavonolignans [34]. Sonication reduced the degradation of the flavonolignans and improved the determinations in milk thistle seeds and finished products.

### Single-laboratory validation

#### Selectivity

Due to the complex mixtures of components in milk thistle extracts, resolution of >1.0 was achieved for five of the major flavonolignans from interfering peaks. Silydianin contained a small co-eluting component at the front of the peak, for which resolution could not be improved; therefore, resolution of less than 1.0 was obtained for this peak in the test samples. There was no evidence of chromatographic interferences at 288 nm from other botanical sources in the multicomponent formulations such as schizandra berry, dandelion extract, or artichoke extract.

#### Linearity

The calibration curves constructed throughout the optimization and validation studies were linear based on visual inspection. The correlation coefficients were above 0.998, confirming the linearity of the analytical range for the individual flavonolignans.

#### Limits of detection and quantitation

Variance checks showed that the method used was applicable for the analytes. The MDL and LOQ for each of the flavonolignans are reported in Table 4.

**Table 4:** Method detection limit (MDL) and limit of quantitation (LOQ) calculated for each of the flavonolignans

Flavonolignan	MDL (μg/mL)	LOQ (μg/mL)
Silychristin	0.01	0.27
Silydianin	0.05	0.14
Silybin A	0.03	0.08
Silybin B	0.06	0.15
Isosilybin A	0.02	0.06
Isosilybin B	0.03	0.07

#### Precision

The responses for all of the analytes in the test materials were above the methods limit of detection. The silydianin peak was below the limit of quantitation in two tincture products; therefore, it was reported solely as detected and no precision analysis was performed for silydianin in these products. The results from three separate days of analysis indicated that all of the analytes had adequate precision in all of the matrixes evaluated. The HorRat values for the raw materials ranged from 0.86 to 1.55, dry finished products ranged from 0.27 to 1.48, and liquid finished products ranged from 0.21 to 0.98. These values are within the acceptable range stated by the AOAC SLV guidelines for dietary supplements [26]. The precision data for raw materials and dry finished products has been summarized in Table 5 and for liquid finished products in Table 6.

**Table 5:** Precision results summary for milk thistle raw materials and dry finished products

Matrix	Analyte	Mean (%*w*/*w*)	HorRat	RSD (%)
MT-RM001	Silychristin Silydianin Silybin A Silybin B Isosilybin A Isosilybin B	0.26 1.04 0.20 0.30 0.21 0.14	1.05 1.32 1.10 1.35 1.07 0.93	5.17 5.23 5.58 6.49 5.41 4.96
MT-RM002	Silychristin Silydianin Silybin A Silybin B Isosilybin A Isosilybin B	0.37 1.07 0.29 0.45 0.25 0.16	1.00 1.55 1.13 1.15 1.05 0.86	4.65 6.13 5.44 5.21 5.19 4.56
MT-BE001	Silychristin Silydianin Silybin A Silybin B Isosilybin A Isosilybin B	13.54 0.38 13.28 19.35 4.75 1.56	0.58 0.77 0.58 0.42 0.36 0.27	1.57 3.57 1.58 1.07 1.13 1.03
MT-CP001	Silychristin Silydianin Silybin A Silybin B Isosilybin A Isosilybin B	4.80 2.33 3.56 5.46 2.01 0.89	0.86 1.48 1.07 0.84 0.71 0.84	2.71 5.21 3.52 2.59 2.56 3.44
MT-CP002	Silychristin Silydianin Silybin A Silybin B Isosilybin A Isosilybin B	5.46 4.49 6.14 8.98 2.84 1.37	0.96 1.02 1.23 1.32 0.95 1.20	2.97 3.24 3.74 3.78 3.24 4.59
MT-CP003	Silychristin Silydianin Silybin A Silybin B Isosilybin A Isosilybin B	6.51 9.85 6.61 10.17 4.07 2.23	0.45 0.60 0.57 0.68 0.65 0.58	1.35 1.70 1.72 1.92 2.10 2.04
MT-TB001	Silychristin Silydianin Silybin A Silybin B Isosilybin A Isosilybin B	0.33 0.09 0.31 0.47 0.14 0.06	0.44 1.31 0.33 0.47 0.34 0.84	2.09 7.56 1.59 2.13 1.83 5.19
MT-TB002	Silychristin Silydianin Silybin A Silybin B Isosilybin A Isosilybin B	2.45 2.09 2.35 3.56 1.22 0.61	0.43 0.35 0.4 0.87 0.44 1.20	1.51 1.26 1.41 2.86 1.72 5.15

**Table 6:** Precision results summary for milk thistle liquid finished products

Matrix	Analyte	Mean (μg/mL)	HorRat	RSD (%)
MT-TN001	Silychristin Silydianin Silybin A Silybin B Isosilybin A Isosilybin B	356.5 detected 198.5 286.3 112.3 28.8	0.35 n/a 0.70 0.49 0.36 0.33	2.32 n/a 5.07 3.34 2.83 3.20
MT-TN002	Silychristin Silydianin Silybin A Silybin B Isosilybin A Isosilybin B	7.64 detected 7.88 10.60 5.75 4.29	0.84 n/a 0.84 0.65 0.31 0.45	9.85 n/a 9.88 7.28 3.80 5.75
MT-TN003	Silychristin Silydianin Silybin A Silybin B Isosilybin A Isosilybin B	367.8 180.4 255.6 407.3 219.7 139.9	0.21 1.00 0.87 0.44 0.46 0.35	1.39 7.31 6.04 2.84 3.28 2.66

#### Accuracy

qNMR was used to confirm the purities provided by the suppliers for the flavonolignan standards. Five of the six standards were found to have residual solvents, with a purity of greater than 98 % without considering the residual solvent amounts. The isosilybin B standard was contaminated with a small organic compound, where two NMR signals were observed in the spectrum. It was concluded that the mass percentage of this component would be low due to the low molecular weight of the contaminant, although the mole percentage of the contaminant was 11 %. Due to the high purities observed in qNMR for the flavonolignans, the purities provided by the suppliers, ranging from 94–96 % including residual solvent levels, were used for quantitation.

The silybin A/B mixed standard was assessed for purity in comparison to the individual silybin A and silybin B reference standards. The calculated purity of silybin A was 47.2 % and for silybin B it was 50.2 %. The values obtained were slightly different in comparison with the suppliers’ specifications of 47.0 and 52.6 % respectively, although the total silybin content is similar (97.4 % calculated versus 97.1 % supplier specifications). The silybin A/B mixture is a suitable alternative to be used in place of the individual reference standards silybin A and B.

The method accuracy was evaluated using a negative control spike recovery test at three concentration levels for all six flavonolignans. The spike concentrations ranged from 1.5 to 11.8 % *w*/*w* total flavonolignans in order to cover the concentrations found in milk thistle seeds and capsules, while the individual flavonolignan ratios were similar to those found in milk thistle seeds. The results for recovery are summarized in Table 7, where the average ranged from 94.6 to 99.9 %.

**Table 7:** Negative control spike recovery results for individual flavonolignans at three spike concentrations

Standard	Spiked concentration (ppm)	Recovery (%)	RSD (%)	Average recovery (%)
Silychristin	10	90.3	2.96	94.6
	35	99.3	2.33	
	90	94.0	1.57	
Silydianin	5	92.5	3.54	95.8
	50	97.9	1.95	
	90	97.0	1.10	
Silybin A	10	90.2	1.26	95.6
	30	99.2	2.64	
	90	97.4	2.19	
Silybin B	25	98.3	2.75	97.5
	50	98.2	3.53	
	120	96.1	2.72	
Isosilybin A	5	95.6	3.85	95.9
	25	94.6	3.14	
	50	97.3	2.64	
Isosilybin B	5	101.8	11.85	99.9
	10	95.4	8.60	
	30	102.5	4.97	

#### Stability of standards

Throughout the validation, samples were in queue for hours prior to injection; therefore, a mixture of flavonolignan standards in methanol was stored at room temperature for 72 h to confirm flavonolignan stability. Based on the minimal changes in peak area (<2 %) from the initial injections, no degradation of the flavonolignans occurred. It is recommended that stock solutions be stored at −20 °C when not in use, as no long-term storage analysis was performed at higher temperatures.

## Conclusions

The INA method for flavonolignan quantitation recommended by the AOAC ERP underwent significant redevelopment and optimization according to statistically guided optimization procedures. The optimized method used a simple and fast sample preparation procedure and was comparable to the original method. The method was subjected to a single-laboratory validation according to AOAC International SLV guidelines for milk thistle raw materials, bulk extracts, dry finished products, and tinctures. All of the parameters investigated were found to be within compliance with the guidelines. Therefore, this method is suitable for determining the concentration of the 6 main flavonolignans silychristin, silydianin, silybin A, silybin B, isosilybin A, and isosilybin B in single component milk thistle preparations or in combination with dandelion, schizandra berry, and/or artichoke extract. It is recommended that this method be adopted as Official First Action method status by AOAC International.
